# Development and Refinement of a Matrix Competency Framework, with Associated Entrustable Professional Activities, to Support Initial Pharmacy Education in Kuwait

**DOI:** 10.3390/pharmacy11050149

**Published:** 2023-09-19

**Authors:** Pierre Moreau, Mohammad Qaddoumi, Dalal Al-Taweel, Sarah Alghanem, Tania Bayoud, Maryam Alowayesh, Monerah Al-Soraj, Mohsen Hedaya, Asmaa Al-Haqan, Danah Alsane

**Affiliations:** College of Pharmacy, Health Sciences Center, Kuwait University, P.O. Box 24923, Safat 13110, Kuwait; pierre.m@ku.edu.kw (P.M.); mohammad.qaddoumi@ku.edu.kw (M.Q.); sara.alghanem@ku.edu.kw (S.A.); tania.bayoud@ku.edu.kw (T.B.); maryam.alowayesh@ku.edu.kw (M.A.); monerah.alsoraj@ku.edu.kw (M.A.-S.); mohsen.hedaya@ku.edu.kw (M.H.); asmaa.alhaqan@ku.edu.kw (A.A.-H.); danah.alsane@ku.edu.kw (D.A.)

**Keywords:** competency-based, pharmacy, education, matrix competency framework, Kuwait, entrustable professional activities (EPA)

## Abstract

The development of competency frameworks serves as the foundation for the development of competency-based education. It is vital to develop a country-specific framework to address the specific needs of the local population for pharmacy services. This study aimed to describe the development process of a competency framework for undergraduate pharmacy education in Kuwait with a unique matrix structure. The process started with the development of guiding principles for curriculum revision and implementation, as well as the identification of global educational outcomes. This process was followed by: (A) a needs assessment with key stakeholders; (B) development of the initial competency framework; and (C) refinement of the framework. Qualitative data were thematically analyzed to identify the main competency domains that students need to perform the identified entrustable professional activities (EPAs). Five population needs were identified by the needs assessment, with 17 EPAs suggested to fulfill those needs. In addition, 11 competency domains were identified. The initial competency framework was created as a 3 × 8 matrix, with 3 professional and 8 transversal competency domains. Refinement of the framework resulted in the removal of redundancies and the development of a global behavior competency profile. The development of a matrix competency framework and associated EPAs for Kuwait serves as a foundation for preparing pharmacists to fulfill local population needs and expanding the scope of practice in the country.

## 1. Introduction

To support the provision of competent pharmacists, the International Pharmaceutical Federation (FIP) proposed a needs-based education model to guide colleges of pharmacy in developing a competency framework that would be aligned with local needs [1,2]. The model suggests that those needs of the population are to be met by relevant professional activities (or services) performed by pharmacists. Pharmacists must be competent in providing those services, and these competencies should drive the education curriculum [3]. The development of competency-based curricula, grounded in local needs, has been highlighted in the literature as one of six strategies to meet patient care and education needs [4,5]. It is also considered a means to integrate local health needs with professional priorities by moving away from more traditional education approaches [6].

**Figure 1:**
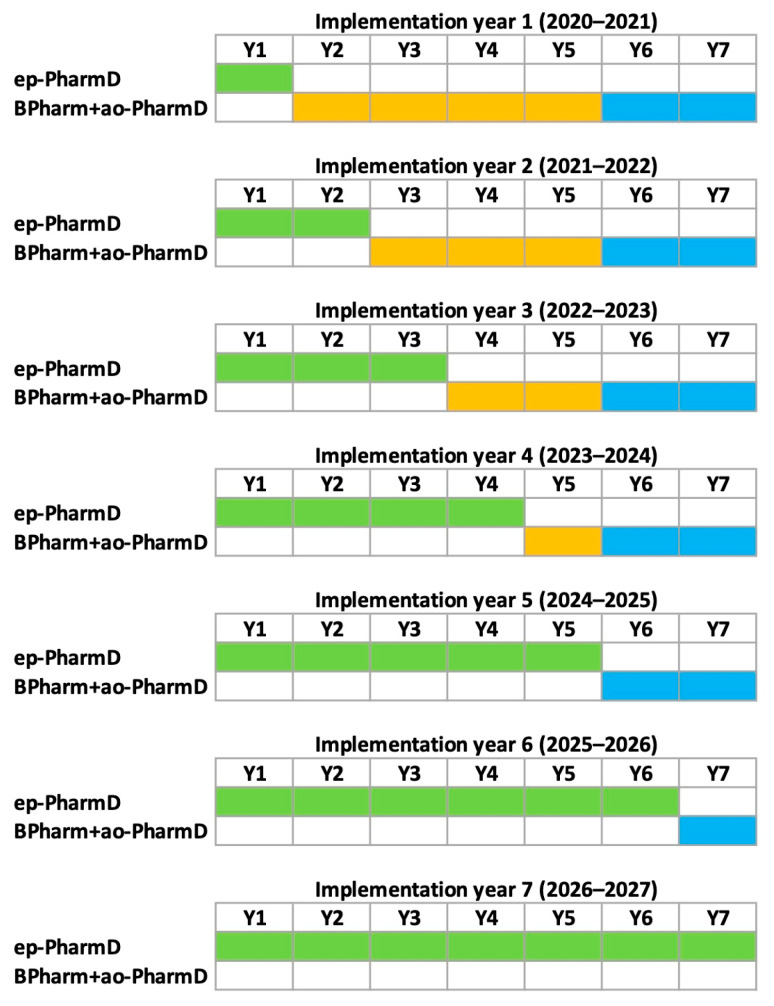
Timeline of the transition between the Bachelor of Pharmacy (BPharm, orange boxes) and two-year elective add-on PharmD programs (ao-PharmD, blue boxes) to a seven-year entry-to-practice PharmD (ep-PharmD, green boxes) curriculum.

The only college of pharmacy in Kuwait is part of Kuwait University, a comprehensive public university. The college was established in 1996 with four academic departments: pharmaceutical chemistry, pharmaceutics, pharmacology and therapeutics and pharmacy practice. The college admitted its first cohort of students in 1997 to a five-year BPharm program. In 2014, the college embarked on a plan to transform its pharmacy education, to better serve the community and to introduce more advanced (clinical) professional activities, in addition to the more traditional pharmacy roles and responsibilities. Considering the scarcity of advanced clinical pharmacy expertise and the unavailability of consistent advanced pharmaceutical services in the country [7], it was decided to proceed in two steps. The first step was to prepare a critical mass of pharmacists who were qualified to provide a broader range of professional services by developing a two-year post-baccalaureate add-on PharmD program, taken after the five-year BPharm at our college (or the equivalent from overseas accredited pharmacy colleges). This program was implemented in 2016 as an elective program, with cohorts ranging from 8 to 24 students. In addition to preparing qualified pharmacists with advanced skills, the add-on PharmD program also aimed to provide the preceptor capacity needed to support the experiential training of students in the next step, which was the development of an entry-to-practice PharmD (ep-PharmD) program. The ep-PharmD, a competency-based program, started in 2020 with cohorts of 70 students, now increased to 100 students in 2023/2024. This ep-PharmD program will slowly phase out the BPharm program and will be the only undergraduate pharmacy degree available at our college (Figure 1). The add-on PharmD program will have been offered for more than 10 years, with an expected output of more than 150 PharmD graduates, when the first ep-PharmD cohort enters their final year of advanced rotations. The preceptor pool chosen from those graduates will be already familiar with entrustable professional activities (EPAs) and their methods of assessment, as they were part of the add-on PharmD training, which will make the transition easier [8].

The construction of a custom-made competency framework is the foundation for the development of competency-based learning since it includes what is expected to be undertaken by pharmacists locally [9,10]. Although several frameworks exist, including the FIP global competency framework [2], it has been recognized that a single global curriculum would not adequately address the needs of individual countries. Thus, it was important to develop a tailor-made framework for Kuwait, personalized to the professional activities expected to be offered by pharmacists in the country [2,11]. According to the model of EPAs [12], each professional activity requires the enactment of a subset of the competency framework and allows for practice and assessment of this subset with a relevant and observable approach, translating those enabling competencies into clinical practice [13,14]. EPAs were central in the curriculum design and were inserted into campus-based courses to prepare students to practice them further within their hospital and ambulatory-care rotations.

This manuscript describes how a competency framework and associated EPAs were developed for undergraduate pharmacy education in Kuwait and the results obtained, as they could help others to follow the same process in their own context. In addition, we compare our framework’s structure with existing frameworks, as it is presented in a unique compact matrix format. The full process of competency-based curriculum development is beyond the scope of this manuscript and is presented elsewhere [15].

## 2. Materials and Methods

A team, with two representatives from each academic department, chaired by the dean of the college at the time (author PM)—who has vast experience in curricular reform—was assembled to revise a draft add-on PharmD program that was already developed in the years prior to 2014. The team initially prepared guiding principles for the curriculum revision and implementation. It was important to define these guiding principles early in the process to steer the development of the curriculum towards a unified vision [15]. It was agreed to adopt a competency-based learning approach tailored for Kuwait to prepare work-ready pharmacists able to perform several professional activities upon graduation, thus focusing on outcomes in the form of abilities of graduates [9,16,17]. Moreover, five global educational outcomes were identified to form the vision of the graduate: 1. patient-centered, collaborative healthcare provider; 2. self-guided, lifelong learner using evidence to support decisions and practice; 3. accountable practitioner with reputable professionalism and ethical behavior; 4. community-oriented professional conceiving value-added services and public health promotion activities; and 5. engaged professional leader and advocate with significant mentoring and communication skills. To support competency-based learning in Kuwait, a competency framework had to be developed, and it involved three steps: (A) a needs assessment (including the identification of EPAs); (B) development of the initial competency framework; and (C) refinement of the framework and simplification of professional activities.

(A) Needs assessment

The objective of the needs assessment was to determine the global population needs with regard to the pharmacists’ contributions, the type of professional services that should be offered to meet the needs (which became our initial EPAs) and the competencies needed to provide professional services. A qualitative study design, using focus group interviews, was used to conduct the needs assessment. Eight focus group interviews were conducted with different groups of stakeholders: pharmacy students, hospital pharmacists, ambulatory clinic pharmacists, industrial and regulatory pharmacists, community pharmacists, pharmacy service managers, physicians and patients. Participants were invited to the interview and only attended if they agreed to be there, and they had the freedom to answer or reject any question. The participants (*n* = 39) were grouped according to their sector and interviewed for approximately 90–120 min to collect data that would provide a deeper understanding from the population of interest. The questions presented in Table 1 formed our interview guide and were predetermined and sequenced to be more logical and understandable to the participants in the focus groups. Each focus group session started with an introduction of the objectives and defining what competencies are. The group interviews were not audio recorded; however, notes were taken by two separate researchers, and a debriefing session was conducted after the end of every group interview to compare notes and any observations noted [18]. The names of the participants were not associated with the answers noted. Each interview started with a blank document of open-ended questions and did not build on answers from prior groups. Data gathered from each group interview were thematically analyzed, and the occurrences of similar words or phrases for needs, professional services, competencies and barriers were counted. It is worth noting that note takers were allowed to ask questions during the sessions to clarify discussion points about needs, services, competencies and barriers and to ensure that they captured the right information when they were unsure. This study provides a snapshot of part of the curricular review process that our faculty undertook, in line with Kuwait University’s vision to improve pharmacy education in Kuwait. It involved the use of non-sensitive, completely anonymous interview procedures (the participants were not defined as “vulnerable”, as participation was not seen to induce any undue psychological stress or anxiety, and no identifiable information was gathered from any participants at any time). All academic staff involved abided by the Kuwait University Code of Ethics when interacting with students [19].

(B) Initial competency framework

The team convened to discuss the results of the needs assessment and start constructing the competency framework. The occurrence of competency domains was calculated by adding them from each set of focus group notes. A competency could be mentioned within several professional activities, explaining why the occurrences could surpass the number of focus groups. For example, the “communication” competency domain could have been brought up for five different professional activities during one focus group. These five occurrences were added to the occurrences in the other focus groups to obtain a total number. Starting from the list of competence domains obtained, a series of competency frameworks and national pharmacy standards of practice were reviewed to identify the enabling competencies (also called competency elements or behaviors) that would define the expected outcomes. The frameworks included the AACP’s Center for the Advancement of Pharmacy Education (CAPE) [20], the Pharmaceutical Society of Ireland (PSI) [21], the National Association of Pharmacy Regulatory Authorities (NAPRA) [22] and the FIP global competency framework [2].

The competency framework was then used to develop competency profiles (subsets of enabling competencies) for each of the professional activities initially identified during the needs assessment. These professional activities, with their unique competency profiles, became the EPAs that were integrated as the backbone of the add-on PharmD curriculum. For each EPA, the competency profile was used to determine the learning activities to be performed, the assessment criteria and the rotations’ learning objectives. The level of entrustment was used to assess the students, as previously published [23]. When combined, all EPAs used each enabling competency at least once, with some enabling competencies used by several EPAs.

(C) Refinement of the competency framework

In 2019, after three years of using the initial framework and EPAs in the add-on PharmD program and in preparation for the launch of the ep-PharmD, the EPAs were critically revised and further simplified. This goal was accomplished by a task force comprised of educators who were involved in the add-on PharmD and, more specifically, in competence assessments using the initial EPA profiles. The first task was to reduce redundant EPAs and combine those that had similar competency profiles. Then, rather than starting from the competency framework itself, the task force worked from the competency profiles and identified enabling competencies that were either redundant, needed reformulation or were difficult to assess during EPA performance. Changes in the profiles were reflected in the global competency framework, and this revised framework is presented (see results).

## 3. Results

(A) Needs assessment

**Figure 2:**
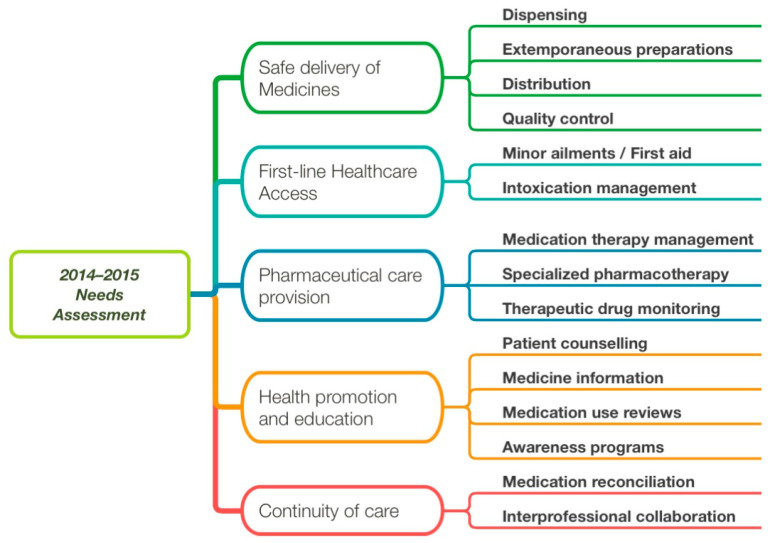
Relations between population needs (rounded boxes) and professional activities (provided or expected) as expressed in the 2015 focus groups. Professional activities were discussed within a population need, hence their hierarchical representation. Those professional activities were used in the form of EPAs within the curriculum.

During the needs assessment, five population needs were identified as met, unmet or future needs (Table 2). According to the sequence of questions (Table 1), the stakeholders then further defined each need in terms of current or expected pharmaceutical professional activities or services. In total, 17 professional activities were suggested within the five population needs (Figure 2) and became our initial EPAs. Table 3 summarizes them with the number of times that they were reported in the focus groups, while Figure 2 presents the relationships between population needs and professional services.

The focus group structured questionnaire allowed for the identification of major competency domains that needed to be acquired by students to perform the expected professional services (EPAs). Table 4 shows the number of occurrences of competency domains required for delivering the identified professional activities. Table 5 presents a small sample of answers that were recorded, structured according to the initial question sequence presented in Table 1.

(B) Initial competency framework

While regrouping the competencies that were highlighted by the groups consulted (Table 4), the team identified three professional competency domains: pharmaceutical patient-centered care, health promotion and education and practice management. The other eight competency domains were considered transversal. By creating a 3 × 8 matrix, enabling competencies could be expressed in terms of both their relevance to the professional domain and the transversal domain. For instance, when a student performs a professional activity (EPA) such as medication therapy management, he or she must demonstrate competence in both transversal (e.g., communication) and professional (e.g., pharmaceutical care) competency domains at the same time, as they are intertwined. During this part of the framework construction, it appeared that most published frameworks consulted (see Methods) had redundant enabling competencies due to their linear structure. For example, communication was detailed in terms of enabling competencies, as it constitutes a major transversal competency, but communication skills were also part of delivering direct patient care, managing operations and providing educational activities, which are professional competencies [22]. Thus, communication was expected to be developed both as a stand-alone competency domain and as part of enabling competencies in the practice of pharmacy activities. This finding prompted the idea of developing this matrix competency framework, which intersects with the professional with the transversal competency domains to define the enabling competencies. This approach allows for the inclusion of transversal competency development as part of professional competency development, providing the professional context (Table 6). Published competency frameworks were studied to determine the enabling competencies to be included in each “cell” of the matrix. The NAPRA competency framework, developed in Canada, was intensively used, as it had the greatest coherence with the competency domains identified during our focus groups. In total and after several simplification iterations aimed at reducing redundancies from source frameworks, 77 enabling competencies were identified within the framework (Table 6).

(C) Refinement of the competency framework

**Figure 3:**
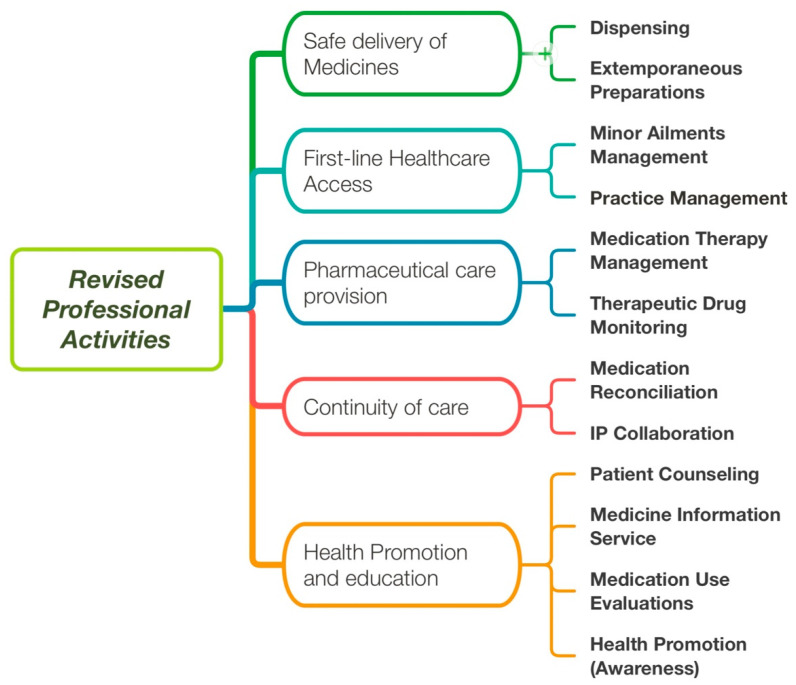
Revised professional activities (used as EPAs) after years of experience with the previous list (Figure 1). IP: Interprofessional.

After three years of using the initial framework, it was thoroughly revised (see Methods). First, the number of EPAs was reduced to remove redundancies for those sharing nearly identical competency profiles. For example, medication therapy management, specialized pharmacotherapy and intoxication management were all merged to be under medication therapy management. Similarly, distribution and quality control were grouped under the dispensing EPA. An EPA called practice management was added to better assess this important aspect of practice, which was not captured initially as a professional activity. The number of EPA was reduced from 17 (Figure 2) to 12 (Figure 3), within the same five populations needs.

Additionally, based on experience with the initial framework and as described in the Methods section, the enabling competencies were refined. This additional streamlining step reduced the number of enabling competencies in our competency profile from 77 to 66 (Table 6). Our current revised competency framework is presented in Table 7.

During the competency framework revision, it was noted that some enabling competencies were not assessed as frequently as others. These competencies included the lifelong learning, teamwork (including interprofessional collaboration), professionalism and proactivity (leadership) competency domains. To remedy the situation, a global behavior competency profile was added to the EPAs to capture enabling competencies that are better assessed longitudinally, rather than during the assessed performance of a professional activity occurrence.

## 4. Discussion

Our initial decision to improve pharmaceutical education, first with a two-year add-on PharmD, followed four years later by an ep-PharmD, greatly helped to develop the country’s scope and capacity of advanced pharmaceutical practice. The add-on PharmD graduates are now part of clinical pharmacy units in major hospitals and have introduced a series of professional services supporting pharmaceutical care at multiple practice sites, which have expanded the scope of practice for pharmacists whether at hospitals or in ambulatory care settings. They are also improving the experiential learning capacity for clinical activity supervision. The initial competency framework, with the creation of 17 EPAs and their own competency profiles, allowed us to prepare the add-on PharmD graduates to be work-ready in providing most of these professional activities. Within this two-year add-on program, the same EPAs (and competency profiles) were used when students practiced in our laboratories and during their rotations within the healthcare system. This process allowed for seamless continuity between the first-year (on campus) and the second-year (rotations) of the curriculum. The same model was adopted for the ep-PharmD but with a longer timeframe.

To support competency-based learning, one needs a complete competency framework that includes what is expected to be mastered by pharmacists locally [9,10]. Although several frameworks existed at the time, including a recent publication of the first version of the FIP global competency framework [2], it was decided to develop one for Kuwait, tailored to the professional activities expected to be offered by pharmacists in the country [7]. In hindsight, it was important to perform our own needs assessment, as the environment of pharmacy practice in Kuwait needed a strong evidence-based and progressive vision. The needs assessment was crucial to developing the competency framework, the EPAs and their enabling competency profiles, but it also helped to discuss the evolution of the profession with all stakeholders and to involve the pharmaceutical community in this curricular reform. It provided solid ground to support the improvement of pharmacy education, practice and regulation in the country [24,25]. A survey, performed by Kuwait University in 2018 while preparing the entry-to-practice PharmD, confirmed the results of the 2014–2015 needs assessment and provided additional support to the vision (unpublished information). Interestingly, the barriers identified during the needs assessment focus groups formed the basis of a national workshop that crafted a series of recommendations to advance the scope of pharmacy practice in the country [24]. Thus, a needs assessment serves several purposes and should be considered before any major curriculum revision, even if a local or regional competency framework exists for the profession [1].

The needs assessment also caused us to realize that transversal competencies are generally intertwined with professional activities. The total score for the competency domains presented in Table 4 also became a guide to developing our competency framework and competency profiles for EPAs. The last column of Table 4, which presents the global educational outcomes defined in our guiding principles, shows that there was strong agreement between what the team envisioned in terms of graduates’ abilities (global educational outcomes) and the competency domains identified by our external stakeholders.

As analyzed and reported before, most of the published pharmacy competency frameworks have been shown to be quite similar [26]. However, our framework has the unique feature of being presented in a matrix of professional and transversal domains containing the enabling competencies that can be measured in relevant professional contexts through EPA practice. We believe that this unique structure limits the redundancy very often found in linear frameworks. For instance, in the NAPRA competency framework, the enabling competency 2.1.1 (patient care domain) reads, “Establish and maintain rapport by using effective communication skills”, while the key competence 7.1 (communication and education domain) reads, “Establish and maintain effective communication skills”. This key competency has six enabling competencies that further demonstrate that communication is performed within a professional context. Table 8 below provides a comparison between the structures of different published competency frameworks and the one developed at the College of Pharmacy at Kuwait University (CoP-KU) [20,21,22,27]. Interestingly, frameworks developed for educational purposes (our own and CAPE) seem to have a more limited number of enabling competencies that students are expected to practice and for which they should demonstrate a certain level of competence to become pharmacists. This fact crucially reduced the number of enabling competencies to be measured, as assessing competence at all expected levels of proficiency is a tedious and often rate-limiting endeavor. Other frameworks were developed for practicing pharmacists and included a greater number of enabling competencies that could be a challenge to assess in practice. As suggested by van Loon et al., the number of competencies and EPAs is a delicate balance, and too many details and too much granularity can be pitfalls of using EPAs [13].

The three professional and eight transversal domains of our competency framework are reminiscent of the FIP global competency framework, with our transversal domains being part of the personal/professional cluster and the three other clusters being similar [2,27]. This fact facilitated the transition from the initial education framework (the one presented here) and the continuing professional development framework adapted separately from the FIP global competency framework [25,28]. However, another distinction of our framework is that it has only two levels of descriptors. The column and row headings could be considered competency domains (three professional and eight transversal), and there are more domains than in most frameworks analyzed (see Table 8). Then, the framework presents the measurable enabling competencies directly, without requiring another layer of organization, which is usually provided by describing key competencies (that are not measured). In our framework, this level of organization is afforded by the intersection of the two domains, which provides 24 possibilities, such as communicating while providing patient care or communicating while engaging in health promotions and education. Interestingly, the number, 24, is very similar to the number of key competencies in other frameworks (Table 7).

Having hands-on experience with the first competency framework and measuring student performance though EPAs and their associated competency profiles exposed issues with the wording or the redundancy of several enabling competencies. Indeed, when juxtaposed within a competency profile for an EPA, it is easier to see what represents a unique enabling competency and what is already measured, at least partially, by another competency. Because several competency frameworks are produced by regulatory authorities or associations that do not have hands-on experience with their implementation and measurement, simplification and streamlining may not occur as readily. Such frameworks are often intellectual activities involving several stakeholders, but they are not necessarily revised after testing in the field. We had the advantage of doing so, and it had a significant impact on simplifying, thus improving the use of our learning and assessment tools. It is our contention that, if an enabling competency is not used within a professional activity (as defined in the EPA profiles in our model), then it is theoretical (often centered around knowledge), cannot be effectively measured and should be removed from a competency framework. Indeed, it is most likely implicit within another enabling competency, like knowledge is always [29].

Using EPAs as a means to measure competence makes the process much easier, as the practice and assessment of EPAs can be distributed throughout the curriculum and rotations [6]. However, some enabling competencies are difficult to assess during a single professional activity performance and represent a more longitudinal impression. For example (from Table 7), the enabling competency CP2—“Accept responsibility and accountability for own actions and decisions in relation to patients’ medication therapy needs”—can be more easily assessed during a full rotation than during the single performance of a professional activity, in which this enabling competency may not need to be demonstrated. Several of these enabling competencies were grouped into a competency profile called global behavior, which is assessed in parallel to the EPA’s profile. This competency profile is now used in rotations and in the pharmaceutical sciences and practice laboratories of the ep-PharmD as well to increase the number of occurrences when these enabling competencies are practiced and assessed. It also highlights the importance of such enabling competencies for students during their on-campus years.

### Limitations

The creation of a specific competency framework also bears some limitations. Its applicability to other colleges or countries is limited, especially considering that the societal needs and professional activities (EPAs) identified are likely to differ. However, the objective of this work was to provide a structured process to build a personalized framework and to create a format for a competency framework that limits redundancies. There is no intention to suggest that the competency framework presented here could be considered as a universal framework or that the EPAs that were identified apply globally. In addition, the construction of the framework depends on the knowledge and experience of the people involved, as several decisions to include or exclude enabling competencies can be subjective. The revision of our framework after years of usage clearly illustrates that it is a living document that requires regular revision to stay current and useful to support the advancement of the profession.

## 5. Conclusions

The College of Pharmacy at Kuwait University has successfully developed and implemented a competency framework to support its competency-based learning efforts after assessing the need of the country. Hands-on experience with the initial framework and EPAs allowed for a thorough and practical revision of the framework to improve it further. Efforts were undertaken to remove redundancies, avoid explicit knowledge and skills (they are implicit to competence) and focus on measurable enabling competencies. Thus, the compact matrix-shaped framework has a limited number of enabling competencies that are suitable, with the use of a competency profile for each EPA, for frequent practice and assessment. The needs assessment provided the relevant professional activities (EPAs) and the competency framework (and the competency profiles, representing their intersection), which are the foundations of our educational efforts to prepare pharmacists to fulfill the population’s needs in Kuwait. It was also instrumental in engaging external stakeholders in expanding the scope of practice in the country, based on locally obtained evidence.

## Figures and Tables

**Table 1:** Questions used in the focus groups that formed the main interview guide. Each main question was followed by a series of sub-questions for each need and each service.

Main Question	Sub-Questions
Q1: What are the needs of Kuwait’s population and healthcare system that are currently met by pharmacists and the profession?	Q1.1: What services are currently provided by pharmacists to fulfill this need? Q1.2: What competencies are needed to provide each identified service? Q1.3: What are the barriers to providing each identified service
Q2: Do you think that there are needs of the population (and healthcare system) that have yet to be met by pharmacists in Kuwait?	Q2.1: What services should be provided by pharmacists to address this need? How do you compare the pharmacists and pharmacy services between Kuwait and those you see when you go abroad? Q2.2: same as Q1.2 Q2.3: same as Q1.3
Q3: What other needs do you perceive pharmacists will have to address in the future?	Q3.1: What services could be provided to address this future need? What additional education “domain” should they obtain from the University? Q3.2: same as Q1.2 Q3.3: same as Q1.3

**Table 2:** Number of groups (max *n* = 8) introducing the population need.

Population Need (Met or Unmet)	*n*
Safe delivery of medications	8
Pharmaceutical care provision	7
Health promotion and education	7
Continuity of care	4
First-line healthcare access	3

**Table 3:** Number of groups (max *n* = 8) introducing a professional activity or service within one or more population needs.

Services (Currently Provided or Potential)	*n*
Dispensing	8
Extemporaneous preparation	8
Medication therapy management	7
Medicines information	6
Patient counseling	6
Medicine reconciliation	3
Healthcare professional collaboration	3
Distribution (supply)	2
Physical assessment and minor ailment management	2
Medicine administration	2
First aid and medical devices	2
Therapeutic drug monitoring	2
Specialized pharmacotherapy	2
Health promotion	2
Medication use review	2
Intoxication management	1
Quality control	1

**Table 4:** Number of occurrences of competency domains discussed as required for delivering the current or expected professional activities and their relations to the five global educational outcomes initially set.

Competency Domains	*n*	Global Educational Outcomes *
Communication	33	5—engaged professional leader and advocate with significant mentoring and communication skills
Patient-centered care (incl. medical devices and patient engagement)	18	1—patient-centered, collaborative healthcare provider
Lifelong learning	16	2—self-guided, lifelong learner using evidence to support decisions and practice
Intra- and interprofessional collaboration (teamwork)	12	1
Professionalism	12	3—accountable practitioner with reputable professionalism and ethical behavior
Information access and critical appraisal	11	2
Pharmacy management	10	3
Management (time, HR, workflow, safety, IT, business)	10	4—community-oriented professional conceiving value-added services and public health promotion activities
Problem solving	10	2
Medicine preparation (incl. IV, TPN)	8	1
Proactivity and leadership	6	5
Public services and education	2	4

* As defined in the guiding principles before the needs assessment activity. Each of the five global educational outcomes was detailed once, and its number was used in other instances.

**Table 5:** Examples of information collected during the focus groups, following the question sequence presented in Table 1. Letters in parenthesis refer to the group from which the answer came.

Needs	Services	Competencies	Barriers
Question 1 (met need)			
(S) Provide information to healthcare professionals about new medicines	Documentation of information requests	Lifelong learning	No real drug information center
(HP) Preventing medical errors	Pharmaceutical care	Problem solving/critical thinking Communication Use of technology Management of pharmacy services Leadership	Language issues between education (English) and practice (Arabic) Lack of confidence Lack of practice Information resources not readily available
(PCP) Access to medicines	Provide medication and minimal counseling	Communication skills Conflict management resolution Stock management Time management HR management Leadership	Job description is not clear (from government) Cannot modify a prescription Electronic resources not readily available
Question 2 (unmet need)			
(CP) Pharmaceutical care	Solve drug-related problems	Professionalism and ethics Health promotion Leadership Marketing of services	Rules and regulations are not clear (substitutions, antibiotic use, etc.)
(HP) Health promotion and education	Public education	Teamwork Communication Ethics Social skills	Lack of training and guidance Comes from personal motivation (no incentive)
(HCP) Clinical pharmacy	Being part of the rounding team	Clinical knowledge and practice Teamwork Communication Lifelong learning Professionalism and ethics	Pushback from some physicians Need an expanded scope of practice

S: students; HP: Hospital pharmacist; PCP: Primary care pharmacist; CP: Community pharmacist; HCP: Healthcare professional (other than pharmacists).

**Table 6:** Initial competency framework presented with the number of enabling competencies in each cell (total of 77 competency elements) of the matrix composed of professional (columns) and transversal (rows) competency domains. In parentheses are the changes made in the 2019 revised competency framework, composed of 66 enabling competencies.

Pharmacy Graduates	Provide Pharmaceutical Care	Engage in Health Promotion and Education	Manage Pharmacy Practice
Communicate	6 (−1)	6 (−3)	2
Access and critically appraise information	3	2 (−1)	1
Solve problems and make decisions	7 (−1)	3 (+1)	3
Collaborate	3 (−1)	1	3 (−1)
Engage in lifelong learning	2 (−1)	2 (+1)	1 (−1)
Act professionally	3	2	5
Manage	4 (−2)	2	8 (−1)
Be proactive	2	3	3

**Table 7:** Final competency framework for entry into pharmacy practice. At the end of their ep-PharmD program, pharmacists.

	**Provide Pharmaceutical Care** *(Pharmacists, in partnership with the patient and caregivers and in collaboration with other HCPs, meet patients’ health and medication-related needs to achieve patients’ health goals)*	**Engage in Health Promotion and Education** *(Pharmacists use their expertise to advance the health and wellness of patients, communities and populations)*	**Manage Pharmacy Practice** *(Pharmacists ensure accurate product distribution and oversee the practice setting with the goal of ensuring safe, effective and efficient patient care)*
**Communicate** *(Pharmacists communicate effectively with patients, the pharmacy team, other HCPs and the public, providing education when required using effective verbal, non-verbal, listening and writing skills in both individual and group settings)*	CC1—Develop and maintain a professional relationship with the patient using effective communication and having a caring attitude (empathy, sensitivity, respect and cultural awareness) CC2—Use appropriate interview techniques to assess patients’ health, needs and goals for their medication therapy (incl. cultural dimensions, involvement in own health, daily activities) CC3—Obtain all needed information using appropriate resources CC4—Communicate the rationale for the MTP (including the monitoring plan) with the patient and healthcare team to favor their involvement CC5—Address non-adherence issues using constructive and motivational approaches	EC1—Incorporate health promotion dimensions into patients’ MTPs and counseling, including responsible use of medicines EC2—Adapt the communication strategy and techniques for the needs of the target audience, including appropriate technology and visual aids EC3—Clarify personal points of view assertively using appropriate arguments	MC1—Manage interpersonal interactions, including conflicts MC2—Use communication techniques to maximize safety by focusing on recipient understanding
**Access and Critically Appraise Information** *(Pharmacists access, retrieve and critically analyze and apply relevant information to make evidence-informed decisions within their practice and management with the goal of ensuring safe and effective patient care)*	CA1—Assess and interpret the relevance, accuracy and completeness of the information in relation to patients’ needs CA2—Identify and prioritize current and potential drug therapy problems (DTPs) CA3—Assess the effectiveness and safety of the implemented MTP, including patients’ needs, adherence and capacity to self-administer medicines	EA1—Identify and critically analyze varied, appropriate and scientifically sound sources of information to formulate rational explanations	MA1—Select the appropriate products and ingredients for the situation based on evidence (bio-equivalency, therapeutic equivalency, quality)
**Solve Problems and Make Decisions** *(Pharmacists access, retrieve and critically analyze and apply relevant information to make evidence-informed decisions within their practice and management with the goal of ensuring safe and effective patient care as a medication therapy expert)*	CS1—Recommend laboratory testing to provide needed information CS2—Perform or request physical assessments to complete needed information CS3—Apply first aid when required CS4—Perform appropriate patient-specific calculations CS5—Identify possible evidence-based treatment options and recommend optimal patient MTPs (pharmacologic, non-pharmacologic, complementary and alternative medicines, devices, route of administration, therapeutic equivalency, cost) CS6—Revise the MTP according to the assessment of effectiveness, safety and adherence	ES1—Write and provide clearly and proficiently relevant scientific arguments for specific contexts and target audiences ES2—Minimize the risk of disease transmission within the practice setting ES3—Promote the reporting (or report) adverse drug reactions (ADRs) appropriately to the relevant authority ES4—Apply the principles of scientific inquiry and decision-making frameworks using evidence to formulate rational explanations	MS1—Select, obtain and record appropriate ingredients and excipients to prepare medicines MS2—Perform pharmaceutical preparations or compounding from master formulas and according to established standards (sterile and non-sterile) MS3—Develop and implement solutions and services to address problems or needs in pharmacy practice, aligned with the principles of patient safety
**Collaborate** *(Pharmacists work in collaboration with the pharmacy team, other HCPs and groups/associations to deliver comprehensive services, make best use of resources and ensure continuity of care to achieve patients’ health goals)*	CX1—Understand, recognize and respect the roles and responsibilities of each party on the healthcare team for appropriate referral CX2—Participate in the assessment of patients and the development and follow up of a collaborative care plan	EX1—Collaborate in the development and implementation of health promotion strategies, public health campaigns and patient safety initiatives	MX1—Address concerns related to prescriptions (validity, clarity, appropriateness, completeness, authenticity) MX2—Contribute to effectiveness in collaborative teams (role clarification, goal setting, resources, responsibilities, accountability, conflict management, feedback)
**Engage in Lifelong Learning** *(Pharmacists seek to remain competent and update their knowledge, skills and attitudes to offer optimal education and patient care throughout their professional career)*	CL1—Use real-life situations to evaluate their practice and update knowledge to offer the best treatment options to the patients and to manage patients with unfamiliar medication-related needs	EL1—Keep abreast of emerging medication safety information EL2—Use the introduction of new medicines, devices and diseases to provide patient, HCP and population education EL3—Identify early the potential of new discoveries to improve the pharmaceutical care of patients	
**Act Professionally** *(Pharmacists uphold self-regulation within legal requirements, professional standards of practice, codes of ethics and policies for the fulfillment of their professional obligations)*	CP1—Apply appropriate judgment and methods to involve and share documentation with other HCPs or caregivers (confidentiality) CP2—Accept responsibility and accountability for own actions and decisions in relation to patients’ medication therapy needs CP3—Ensure that patient safety and quality of care are at the center of their practice (subordinating their personal interests), while maintaining appropriate boundaries	EP1—Apply integrity in practicing and advocating for the profession EP2—Advocate for the responsible use of medicines to support optimal use	MP1—Uphold ethical principles in professional conduct and decision making MP2—Practice within legal requirements in terms of standards, safety and confidentiality MP3—Treat colleagues with sensitivity, empathy, respect and dignity MP4—Maintain a professional image and composure in difficult situations MP5—Seek guidance and accept feedback
**Manage** *(Across the different components of their practice, pharmacists apply good management principles for time and human resources management, process optimization and marketing of services)*	CM1—Maintain complete and accurate patient records to document actions, interventions, results and follow-ups to facilitate continuity of care with HCPs CM2—Perform appropriate filling, packaging, labeling and accuracy checks according to patients’ information and needs	EM1—Manage all aspects of in-services, new services development or health promotion projects, including background analysis, planning (incl. business plan), implementation and assessment EM2—Apply the principles of pharmacoeconomic assessment and cost-benefit analysis of medicines	MM1—Practice within the legal requirements for the medicine supply chain (manufacture, supply, collection, storage, usage and disposal) MM2—Oversee inventory management to support safe and efficient dispensing according to official formularies MM3—Optimize and address safety and efficiency issues in the dispensing chain (personnel, technology) MM4—Solve problems and make decisions in an efficient and timely manner by prioritizing work and safety while managing workloads MM5—Organize the return of recalled, unused or expired medicines by patients MM6—Identify pharmacy resource requirements (HR, IT) and hire, train, supervise and evaluate human resources effectively MM7—Evaluate the quality of care and cost-effectiveness of services provided
**Are Proactive** *(Pharmacists initiate or collaborate in developing, implementing and evaluating research, policies, procedures and other activities that promote quality and safety, health and well-being and their profession)*	CV1—Develop and utilize tools and services to aid patients in understanding and adhering to their MTPs CV2—Lead by example for patient care and inspire confidence in patients, peers and other HCP s(role model)	EV1—Contribute to the evolution of policies and guidelines for pharmacy practice with professional organizations EV2—Contribute to the development of new knowledge by engaging in practice-based research activities EV3—Engage in the promotion of pharmacy practice as an essential part of the healthcare team	MV1—Accept leadership roles in team-based environments MV2—Address drug misuse and near misses and learn from errors along the medicine dispensing chain in an effort to improve safety MV3—Contribute to the initiation, development and improvement of clinical pharmacy services

CPD: Continuous professional development; HCP: Healthcare professional; HCT: Healthcare team (includes the patient); MTM: Medication therapy management; MTP: Medication therapy plan (includes monitoring parameters of efficacy and safety, techniques and timelines).

**Table 8:** Comparison of the structure of the competency frameworks used in the construction of the one for the College of Pharmacy at Kuwait University (CoP-KU).

Framework	Competency Domains	Key Competencies	Enabling Competencies (Behaviors)
CAPE [20]	4	15	84
NAPRA [22]	9	34	134
FIP v2 [27]	4	25	123
PSI [21]	6	25	178
CoP-KU	11	24	66

## Data Availability

The data presented in this study are available on request from the corresponding author.
